# Genome Sequences of 25 SARS-CoV-2 Sublineage B.1.1.529 Omicron Strains in Bangladesh

**DOI:** 10.1128/mra.00119-22

**Published:** 2022-03-24

**Authors:** Omar Hamza Bin Manjur, Mokibul Hassan Afrad, Manjur Hossain Khan, Mohabbat Hossain, Zannat Kawser, Ahmed Nawsher Alam, Nandita Banik, Saruar Alam, Mallick Masum Billah, Nawroz Afreen, Farhana Khanam, Taufiqur Rahman Bhuiyan, Mohammed Ziaur Rahman, Emilie Westeel, Jean-Luc Berland, Florence Komurian-Pradel, Sayera Banu, Mustafizur Rahman, Nicholas R. Thompson, Firdausi Qadri, Tahmina Shirin

**Affiliations:** a Institute for Developing Science and Health Initiatives, Dhaka, Bangladesh; b International Centre for Diarrheal Disease Research, Bangladesh, Dhaka, Bangladesh; c Institute of Epidemiology, Disease Control, and Research, Dhaka, Bangladesh; d Fondation Mérieux, Direction Médicale et Scientifique, Lyon, France; e Wellcome Sanger Institute, Wellcome Genome Campus, Hinxton, Cambridgeshire, United Kingdom; f London School of Hygiene and Tropical Medicine, London, United Kingdom; DOE Joint Genome Institute

## Abstract

We report the coding-complete genome sequences of 25 severe acute respiratory syndrome coronavirus 2 (SARS-CoV-2) sublineage B.1.1.529 Omicron strains obtained from Bangladeshi individuals in samples collected between December 2021 and January 2022. Genomic data were generated by Nanopore sequencing using the amplicon sequencing approach developed by the ARTIC Network.

## ANNOUNCEMENT

Severe acute respiratory syndrome coronavirus 2 (SARS-CoV-2) (family *Coronaviridae*, genus *Betacoronavirus*) is a positive-sense single-stranded RNA virus (1). The most recent emerging SARS-CoV-2 variant of concern (VOC), Omicron (B.1.1.529), was first reported in South Africa and Botswana and has gained considerable attention because of its high transmissibility and possible immune escape potential (2). Among the three sublineages of the Omicron variant (BA.1, BA.2, and BA.3), BA.1 and BA.3 possess the characteristic spike (S) gene target failure (SGTF) due to a deletion (Δ69–70) in the primer target site, while the BA.2 viral genome does not possess this deletion (3).

As part of the ongoing SARS-CoV-2 genomic surveillance (protocol IEDCR/IRB/2020/11) by the Institute of Epidemiology, Disease Control, and Research (IEDCR), Bangladesh, two specimens were obtained from individuals who had recently visited Africa and had reported coronavirus disease 2019 (COVID-19) symptoms on return to Bangladesh. These specimens were found to be positive for the SARS-CoV-2 nucleocapsid (N) gene but negative for the S gene by TaqPath COVID-19 Combo reverse transcription (RT)-PCR (Applied Biosystems, Bedford, MA, USA). Seventeen more SARS-CoV-2-positive specimens from the countrywide SARS-CoV-2 surveillance showed similar results. These specimens were further screened with the TaqMan SARS-CoV-2 mutation panel (Applied Biosystems), which indicated the presence of S:N501Y, one of the signature mutations of the SARS-CoV-2 Omicron variant. This S:N501Y mutation is not present in the S gene of the Delta variant, which was the predominant strain circulating in Bangladesh in the last few months of 2021. Overall, a total of 25 specimens were used as input for genome sequencing using the Oxford Nanopore Technologies sequencing platform. The detailed information for all 25 individuals is presented in Table 1.

**TABLE 1:** Data for Bangladesh SARS-CoV-2 Sublineage B.1.1.529 isolates

Sample	Date of sample collection (day/mo/yr)	Patient age (yr)	Sex^*a*^	Symptom(s)^*b*^	Name of vaccine received	Travel history outside Bangladesh	Pangolin lineage	Genome size (bp)	Coverage (%)^*c*^	GC content (%)	SRA accession no.	GenBank accession no.
OIS-0630	6/12/2021	31	F	+	AstraZeneca	Zimbabwe	B.1.1.529.1	29,743	99.5	40.7	SRR17901911	OM570259
OIS-0631	6/12/2021	21	F	+	AstraZeneca	Zimbabwe	B.1.1.529.1	29,743	99.5	42.3	SRR17901910	OM570260
OIS-0633	8/12/2021	47	F	−	AstraZeneca + Pfizer-BioNTech (booster dose)	Egypt	B.1.1.529.1	29,740	99.5	40.2	SRR17901899	OM570261
OIS-0648	14/12/2021	18	F	+	NA	United Kingdom	B.1.1.529.1	29,743	99.5	41.40	SRR17901893	OM570268
OIS-0634	19/12/2021	30	F	−	AstraZeneca		B.1.1.529.1	29,740	99.5	41.30	SRR17901892	OM570263
OIS-0637	19/12/2021	84	M	+	Moderna		B.1.1.529.1	29,743	99.5	40.10	SRR17901891	OM570264
OIS-0649	22/12/2021	23	F	+	Pfizer-BioNTech	United Kingdom	B.1.1.529.1	29,743	99.5	40.60	SRR17901890	OM570269
IR-1955	23/12/2021	56	M	+	Pfizer-BioNTech	Denmark	B.1.1.529.1	29,743	99.5	41.10	SRR17901889	OM570262
OIS-0664	26/12/2021	65	M	NA	Pfizer-BioNTech		B.1.1.529.1	29,743	99.5	40.70	SRR17901888	OM570270
OIS-0666	26/12/2021	53	F	NA	NA		B.1.1.529.1	29,743	99.5	40.00	SRR17901887	OM570271
IR-1979	27/12/2021	49	F	+	Pfizer-BioNTech	Denmark	B.1.1.529.1	29,743	99.5	40.30	SRR17901909	OM570265
IR-1982	27/12/2021	65	M	+	AstraZeneca		B.1.1.529.1	29,743	99.5	40.40	SRR17901908	OM570266
IR-1983	27/12/2021	65	F	+	AstraZeneca		B.1.1.529.1	29,743	99.5	42.10	SRR17901907	OM570267
TND-07-0639	28/12/2021	35	F	+	Sinopharm		B.1.1.529.1	29,743	99.5	40.80	SRR17901906	OM570275
OIS-686	30/12/2021	61	M	+	AstraZeneca		B.1.1.529.1	29,743	99.5	40.70	SRR17901905	OM570272
TND-04-0674	2/1/2022	64	M	NA	AstraZeneca		B.1.1.529.1	29,743	99.5	41.60	SRR17901904	OM570276
TND-04-0675	2/1/2022	29	F	+	Pfizer-BioNTech	Germany	B.1.1.529.1	29,743	99.5	40.40	SRR17901903	OM570277
OIS-688	3/1/2022	48	F	NA	NA		B.1.1.529.1	29,743	99.5	40.10	SRR17901902	OM570273
IR-2027	3/1/2022	35	F	+	AstraZeneca	UAE	B.1.1.529.1	29,743	99.5	40.80	SRR17901901	OM570274
TND-09-0338	6/1/2022	49	M	+	AstraZeneca		B.1.1.529.2	29,729	99.5	41.10	SRR17901900	OM570278
TND-05-0426	8/1/2022	60	M	+	Sinopharm		B.1.1.529.2	29,729	99.5	40.30	SRR17901898	OM570279
TND-04-0735	9/1/2022	19	M	+	Sinopharm		B.1.1.529.2	29,729	99.5	40.20	SRR17901897	OM570280
TND-04-0736	9/1/2022	43	M	+	Sinopharm		B.1.1.529.2	29,729	99.5	40.50	SRR17901896	OM570281
TND-04-0747	10/1/2022	32	M	+	AstraZeneca		B.1.1.529.2	29,714	99.5	40.90	SRR17901895	OM570282
TND-04-0748	10/1/2022	45	M	+	Sinopharm		B.1.1.529.2	29,729	99.5	41.10	SRR17901894	OM570283

a F, female; M, male.

b +, present (fever, cough, or mild weakness); −, absent; NA, information not available.

c With reference to the Wuhan Hu-1 genome (GenBank accession number NC_045512.2).

Viral RNA was extracted from nasopharyngeal swab samples using the QIAamp viral RNA minikit (Qiagen). Sequencing libraries were prepared using the multiplex PCR amplicon sequencing approach developed by the ARTIC Network (4, 5). Libraries were multiplexed and sequenced on an FLO-MIN106D flow cell (R9.4.1) for at least 6 h. Raw reads were base called and demultiplexed with MinKNOW v21.02.1. Processed reads were assembled using the artic gupplyplex script with Medaka v1.4 using the ARTIC EPI2ME v3.3.0 SARS-CoV-2 pipeline (FastQC plus ARTIC plus NextClade) (https://artic.network/ncov-2019/ncov2019-bioinformatics-sop.html). In total, 4,324,431 reads were obtained (range, 79,526 to 745,281 reads per sample; average length, 505 bp). Compared to the reference Wuhan Hu-1 genome (GenBank accession number NC_045512.2), the signature amino acid alterations in the spike protein matching the genetic markers of sublineages B1.1.529.1 and B1.1.529.2 were identified. Among the 25 sequences, Pangolin (github.com/cov-lineages/pangolin) assigned 19 sequences to lineage B.1.1.529.1 (BA.1), and six strains were found to be lineage B.1.1.529.2 (BA.2) (Table 1). These six BA.2 strains were SARS-CoV-2 S gene and S:N501Y positive by SARS-CoV-2 RT-PCR and TaqMan mutation PCR, respectively. SARS-CoV-2 lineage BA.2 lacks the characteristic SGTF-causing deletion (Δ69–70) by conventional quantitative PCR (qPCR), compared to BA.1 and BA.3; therefore, qPCR primarily targeting the absence of SGTF for detection of the Omicron variant will be insufficient for monitoring the spread of the Omicron variant. Here, we report the early detection of SARS-CoV-2 Omicron variant sublineages B.1.1.529.1 (BA.1) and B.1.1.529.2 (BA.2) in the Bangladeshi population, which will be helpful for mitigation of the potential fourth wave of COVID-19 in Bangladesh.

### Data availability.

The data from this study can be found under GISAID accession numbers EPI_ISL_7404462, EPI_ISL_7404463, EPI_ISL_8146774, EPI_ISL_8414987, EPI_ISL_8146772, EPI_ISL_8146773, EPI_ISL_8414988, EPI_ISL_8096971, EPI_ISL_8414989, EPI_ISL_8414990, EPI_ISL_8215676, EPI_ISL_8215677, EPI_ISL_8415001, EPI_ISL_8215678, EPI_ISL_8414993, EPI_ISL_8415003, EPI_ISL_8415004, EPI_ISL_8414994, EPI_ISL_8414995, EPI_ISL_9456595, EPI_ISL_9456604, EPI_ISL_9456606, EPI_ISL_9456607, EPI_ISL_9456620, and EPI_ISL_9456621. The Sequence Read Archive (SRA) and GenBank accession numbers are listed in Table 1.
